# AI-Powered Next-Generation Technology for Semiconductor Optical Metrology: A Review

**DOI:** 10.3390/mi16080838

**Published:** 2025-07-22

**Authors:** Weiwang Xu, Houdao Zhang, Lingjing Ji, Zhongyu Li

**Affiliations:** Shanghai Precision Measurement Semiconductor Technology, Inc., Shanghai 210700, China

**Keywords:** optical spectroscopy, Artificial Intelligence (AI), Mueller Matrix Ellipsometry (MME), physics-informed neural network (PINN), tool-to-tool (T2T) matching, multi-task learning

## Abstract

As semiconductor manufacturing advances into the angstrom-scale era characterized by three-dimensional integration, conventional metrology technologies face fundamental limitations regarding accuracy, speed, and non-destructiveness. Although optical spectroscopy has emerged as a prominent research focus, its application in complex manufacturing scenarios continues to confront significant technical barriers. This review establishes three concrete objectives: To categorize AI–optical spectroscopy integration paradigms spanning forward surrogate modeling, inverse prediction, physics-informed neural networks (PINNs), and multi-level architectures; to benchmark their efficacy against critical industrial metrology challenges including tool-to-tool (T2T) matching and high-aspect-ratio (HAR) structure characterization; and to identify unresolved bottlenecks for guiding next-generation intelligent semiconductor metrology. By categorically elaborating on the innovative applications of AI algorithms—such as forward surrogate models, inverse modeling techniques, physics-informed neural networks (PINNs), and multi-level network architectures—in optical spectroscopy, this work methodically assesses the implementation efficacy and limitations of each technical pathway. Through actual application case studies involving J-profiler software 5.0 and associated algorithms, this review validates the significant efficacy of AI technologies in addressing critical industrial challenges, including tool-to-tool (T2T) matching. The research demonstrates that the fusion of AI and optical spectroscopy delivers technological breakthroughs for semiconductor metrology; however, persistent challenges remain concerning data veracity, insufficient datasets, and cross-scale compatibility. Future research should prioritize enhancing model generalization capability, optimizing data acquisition and utilization strategies, and balancing algorithm real-time performance with accuracy, thereby catalyzing the transformation of semiconductor manufacturing towards an intelligence-driven advanced metrology paradigm.

## 1. Introduction

As semiconductor manufacturing processes persistently approach fundamental physical limits, nano-scale metrology has emerged as the critical bottleneck determining chip performance, yield, and manufacturability. The rapid transition of integrated circuits from planar to three-dimensional (3D) stacked architectures, characterized by feature sizes shrinking into the Angstrom scale (< 1 nm), layer counts exceeding 200, and the pervasive integration of heterogeneous materials, poses unprecedented challenges to conventional metrology techniques. Current mainstream metrology technologies—including Atomic Force Microscopy (AFM) [1,2], Scanning Electron Microscopy (SEM) and its Critical Dimension variant (CD-SEM) [3], and various optical spectroscopy methods [4,5]—though applicable in specific contexts, face an inherent conflict between simultaneously achieving accuracy, speed, and non-destructiveness.

AFM achieves exceptional sub-nanometer vertical resolution (down to 0.1 nm) by exploiting atomic-level force interactions between the probe tip and the sample surface. This enables direct reconstruction of true 3D topography, granting it a unique value in surface roughness characterization. However, its critical flaw is extremely low throughput. The inherent point-by-point raster scanning mode is time-consuming, often requiring several minutes to tens of minutes to acquire a single image. Probe tip wear causes measurement drift and poses a significant risk of physical damage to soft materials or delicate multilayer structures. Consequently, AFM measurements of structures with high aspect ratios (HARs), such as deep trenches or through-silicon vias (TSVs), suffer from severe distortion at the bottom regions and fall entirely short of meeting modern fabs’ requirements for high-volume, in-line, full-wafer inspection throughput.

SEM, particularly its specialized CD-SEM variant tailored for metrology, utilizes a focused electron beam for imaging. It achieves sub-nanometer spatial resolution (0.5–1 nm), enabling the resolution of features like sub-10 nm line widths and Oxide-Nitride-Oxide (ONO) layer thicknesses within devices such as 3D NAND, with reported precision down to 0.08 nm [6]. CD-SEM’s core strength lies in its direct imaging capability of patterned wafers. Critically, however, it is an inherently destructive technique. Electron beam bombardment induces charging effects, altering the electrical properties of devices. More significantly, analyzing cross-sections of multilayer structures (e.g., 200+ layer 3D NAND) necessitates destructive preparation via Focused Ion Beam (FIB) milling, sacrificing the product and eliminating any possibility of in-line, full-wafer inspection. While the use of low-energy electron beams (<1 keV) mitigates some damage, CD-SEM remains hampered by its inherently limited field of view (typically only 10–100 µm^2^) and complex sample preparation requirements. These factors preclude its effective application for rapid, non-destructive, large-area inspection across entire wafers. Although AI has been applied to accelerate CD-SEM image analysis (e.g., increasing defect detection throughput by 16×), these advancements cannot overcome the fundamental limitations of sample destructiveness and finite throughput [7].

In this context, optical spectroscopy techniques—encompassing spectrophotometry and spectroscopic ellipsometry (SE), among others—have become the undisputed cornerstone of semiconductor in-line process control (IPC) and high-volume manufacturing monitoring. This is due to their core advantages: non-contact operation, non-destructiveness, high measurement speed (single-point measurement on the millisecond scale), and large field coverage [8].

Spectrophotometry analyzes variations in the intensity of light reflected from or transmitted through a sample across a broad spectral range. By fitting this data to physical models, such as the Transfer Matrix Method (TMM), it inversely extracts parameters like film thicknesses and optical constants (n, k), achieving sub-nanometer precision (approximately 0.2–0.5 nm) [9,10]. However, it suffers from insufficient sensitivity for highly absorbing media (e.g., metal layers) and inherently acquires only intensity information (amplitude), lacking phase data. This limitation constrains its ability to characterize complex multilayer structures.

Spectroscopic Ellipsometry (SE), conversely, precisely measures changes in the polarization state of light reflected from a sample—specifically the amplitude ratio (Ψ) and phase difference (Δ). By simultaneously acquiring both amplitude and phase information, SE significantly enhances the dimensionality and accuracy of parameter extraction. It achieves thickness measurement precision ranging from 0.1 to 1 nm for dielectric films and can characterize complex optical constant profiles. Consequently, SE has become the predominant technique for monitoring critical parameters such as gate oxide thickness and complex multilayer stacks in in-line applications [11,12].

**Figure 1:**
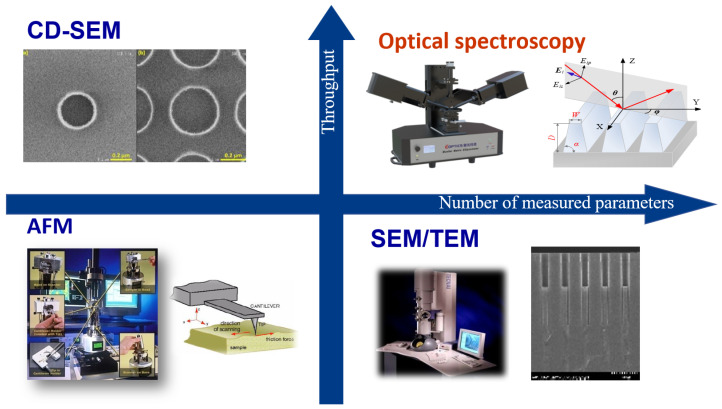
Trade-off analysis of mainstream metrology techniques: X-axis (number of measurable parameters) includes critical dimensions (CDs) and optical constants (n/k values); Y-axis (measurement throughput) shows time efficiency; optical spectroscopy demonstrates superior ability to measure multiple parameters rapidly.

Figure 1 illustrates the trade-offs of mainstream metrology techniques along two key axes: the number of measurable parameters and measurement throughput speed. Optical spectroscopy clearly demonstrates the capability to measure a larger number of critical parameters (CDs, n, and k values) within a shorter timeframe.

However, as semiconductor device structures continue to advance towards increasing three-dimensionality, miniaturization (nano-scale), and complexity, traditional optical spectroscopy is encountering significant challenges, forming three critical bottlenecks.

**Figure 2:**
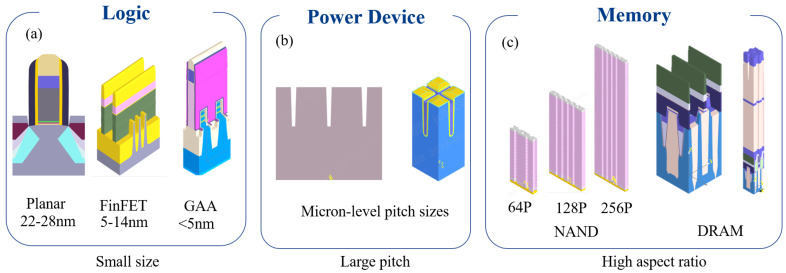
Schematic representation of advanced semiconductor devices: (**a**), logic devices, e.g., Gate-All-Around (GAA) with sub-5 nm features; (**b**), power/RF devices, e.g., IGBT with large-pitch gratings; (**c**), memory devices, e.g., 3D NAND with high-aspect-ratio memory holes.

(1) Scaling Limits for Logic Devices: As shown in Figure 2a, the critical dimensions (CDs) of transistors (such as FinFET gate widths) approach 5 nm (relevant to the 3 nm technology node) [13,14], and the optical diffraction limit (approximately half the wavelength, 200 nm in visible light) severely degrades the signal-to-noise ratio (SNR) of measurement signals. At the nano-scale, optical near-field effects become significant, invalidating traditional far-field optical models. For instance, monitoring the impact of sub-5 nm Line Edge Roughness (LER) and localized deformations on electrical performance requires sub-Angstrom (sub-Å) precision, pushing existing optical tools close to their theoretical sensitivity limits. Furthermore, the oscillation periods in reflectance spectra become extremely dense as dimensions shrink. Consequently, thickness variations of 1 nm often cause reflectance changes of less than 0.1%. Traditional iterative optimization algorithms based on physical models like the Transfer Matrix Method (TMM) or Rigorous Coupled-Wave Analysis (RCWA) are highly susceptible to converging on local minima, leading to significant errors. Additionally, the inevitable nano-scale surface roughness of Atomic Layer Deposition (ALD) films (with root mean square (RMS) roughness values potentially reaching 0.2 nm) introduces additional scattered light, which further interferes with the extraction of the primary signal and exacerbates precision degradation.

(2) Large-Pitch Computation Bottleneck for Power/RF Devices: Power devices (e.g., IGBT, MOSFET ) often utilize large-pitch gratings or superlattice structures to meet requirements for high power and low loss, with periods ranging from several micrometers to tens of micrometers (see Figure 2b). Electromagnetic simulation of such structures relies on rigorous methods like RCWA. As the period size increases, the number of diffraction orders (±100 or more) required for accurate simulation of light-structure interactions (including higher-order diffraction effects and edge field distortions) grows exponentially. This surge in computational complexity (e.g., memory consumption exceeding 10 GB, single simulation times increasing from seconds to hours) renders these approaches entirely impractical for meeting the real-time feedback requirement (second-level response) of in-line metrology. Traditional physics-model-based iterative optimization struggles to converge efficiently within these high-dimensional parameter spaces, becoming excessively time-consuming and prone to local minima. Engineers are often forced to resort to simplified models (e.g., scalar scattering approximations, Effective Medium Theory (EMT)), but this sacrifices accuracy for speed, introducing significant errors (e.g., effective refractive index calculation errors potentially reaching 5%). Compounding this challenge, high thermal conductivity materials commonly used in power chips (like silicon carbide, SiC, and gallium nitride, GaN) often exhibit strong anisotropic optical properties. This anisotropy invalidates traditional scalar approximations, requiring vector electromagnetic modeling and further increasing the computational burden.

(3) HAR-Signal Attenuation for Memory Devices: 3D NAND stack layer counts shown in Figure 2c have surpassed 200 layers [15], with memory hole depths reaching several micrometers while apertures shrink to just tens of nanometers, leading to HAR (>50:1, approaching 100:1). Light propagating within these deep holes undergoes multiple reflections and absorption, resulting in an exponential attenuation of light intensity reaching the bottom (intensity can be attenuated by factors exceeding $10^{4}$ compared to surface reflection). This not only increases measurement errors for bottom-film thickness and morphology (exceeding 5 nm at aspect ratios > 40:1), but also risks completely losing the signal in noise, eliminating the ability to monitor critical bottom layers (e.g., the channel layer, blocking oxide). Furthermore, minor sidewall tilt (taper angle) causes optical path differences, doubling or even quadrupling the oscillation frequency in interference spectra. Traditional models struggle to accurately decouple these highly coupled parameters (taper angle, depth, material refractive index), making data inversion significantly more difficult. Even the use of Extreme Ultraviolet (EUV) spectroscopy (wavelength 13.5 nm) to improve resolution faces significant hurdles: the strong absorption of EUV light by materials causes severe attenuation of phase signals, potentially leading to reconstruction errors exceeding 15%. Additionally, the system cost and the limited penetration capability of EUV through complex multilayer structures remain significant bottlenecks.

Meanwhile, AI technologies, particularly deep learning (DL), have achieved disruptive breakthroughs over the past decade, with their applications deeply penetrating fields such as industrial inspection, predictive maintenance, and complex system modeling. Within semiconductor manufacturing, the successful application of AI has already been validated: Convolutional Neural Networks (CNNs) have achieved 99.9% accuracy in wafer defect pattern recognition [16], significantly surpassing traditional algorithms; and Reinforcement Learning (RL) has been used for real-time parameter tuning in etching and deposition processes, markedly improving uniformity [17]. These practices compellingly demonstrate AI’s capability to handle high-dimensional data and uncover complex nonlinear relationships, offering a novel paradigm for addressing the aforementioned metrology challenges.

The pace of development in metrology equipment has significantly lagged behind that of manufacturing technology innovation. As the industry marches towards the Angstrom scale, thousand-layer stacking, and heterogeneous integration, incremental improvements relying solely on traditional physical models and hardware upgrades struggle to overcome current bottlenecks. Next-generation metrology technology must integrate “smarter algorithms” with “more efficient optical designs.” Mueller Matrix Ellipsometry (MME) emerges as an ideal platform for this convergence [18,19,20]. Compared to conventional SE, which measures only two parameters (Ψ and Δ), MME comprehensively characterizes a sample’s polarization-modulation properties—including depolarization, birefringence, chirality, and more—by acquiring the full 4×4 Mueller matrix ($M_{11}$ to $M_{44}$), thereby increasing the information content by an order of magnitude. This high-dimensional data space offers unique advantages for characterizing advanced structures exhibiting complex optical anisotropy (such as tilted columnar crystals, strained layers) or nano-scale inhomogeneity (e.g., EUV photoresists, self-assembling materials). The dual rotating compensator Mueller matrix imaging ellipsometer (DRC-MMIE) developed by a Chinese research team [21] has achieved spatial resolution better than 40 μm, along with high-throughput measurement capability. Combined with a broadband light source, it enables the simultaneous extraction of material optical constants and thickness distributions, providing a powerful tool for in-line nanostructure metrology and interface characterization of III-V heterostructures.

However, the voluminous data generated by MME (all 16 elements of the matrix) also presents significant analytical challenges—traditional physics-model-based inverse problem solving (e.g., inverting over 1000 parameters for a 200-layer stacked structure) becomes computationally inefficient to the point of impracticality in multilayered, high-dimensional scenarios, often requiring hours to complete. This is precisely where AI can play a pivotal role.

This review aims to provide a overview of the progress and application potential of AI-powered optical spectroscopy in the field of metrology for advanced semiconductor manufacturing. The review will first delve into the fundamental principles and core advantages of cutting-edge optical spectroscopy techniques, with Mueller Matrix Ellipsometry (MME) as a representative example, elucidating its development trajectory and inherent technical bottlenecks in addressing key metrology challenges at the nano-scale, HAR, and complex heterogeneous structures. Subsequently, the article will construct a comprehensive landscape, meticulously reviewing and analyzing the current research status, significant achievements, and existing limitations in the scope of current AI methods (particularly deep learning models) applied across critical stages of semiconductor optical spectroscopy (such as spectral analysis, structure inversion, defect detection, and parameter prediction).

Departing from the core metrology challenges faced by advanced semiconductor manufacturing, this paper introduces the Artificial Intelligence-based Next-Generation Metrology Technology Reference Architecture proposed by Shanghai Precision Measurement Semiconductor Co., Ltd. (Shanghai, China) (PMISH). Serving as a nexus between theoretical analysis and industrial practice, we will provide a specific case study analysis addressing the key technical gaps identified in the preceding discussion. This case study will demonstrate the company’s actual solution based on its innovative architecture, detailing its underlying principles and efficacy in tackling a specific metrology challenge. Consequently, this review establishes a comprehensive application map of AI–optical metrology through systematic categorization of technical approaches into four distinct paradigms: Forward Surrogate Models (Section 3.2); Inverse Prediction Models (Section 3.3); Physics-Informed Neural Networks (Section 3.4); and Multi-stage Network Architectures (Section 3.5). This framework quantitatively demonstrates AI’s transformative capabilities in semiconductor metrology: parameter extraction accuracy reaching sub-nanometer CD precision (e.g., 0.1 nm MAE in grating reconstruction), throughput gains exceeding >100× acceleration vs. traditional methods, and industrial applicability validated through diverse fab-ready solutions. Despite these advances, our analysis reveals persistent research gaps in industrial implementation—particularly in tool-to-tool (T2T) matching consistency. To bridge this gap, we present our PMISH’s proprietary MTL framework as a case study (Section 4), demonstrating how signature decoupling achieves T2T matching on production wafers. This industrial exemplar addresses critical voids in current academic research while maintaining our core technical taxonomy.

## 2. Principles of Optical Spectroscopy Based on MME

MME is an optical measurement technique based on polarization state transformation, used for characterizing the geometric profiles and material properties of nanostructures. Its core principle involves generating incident light with a known polarization state using a Polarization State Generator (PSG). After this light is reflected or transmitted by the sample, the resulting changes in its polarization state are analyzed by a Polarization State Analyzer (PSA). This process is comprehensively described by a 4 × 4 Mueller matrix M, which captures the sample’s complete optical response:

(1) $$\left[\begin{array}{c} I_{o}\\ Q_{o}\\ U_{o}\\ V_{o} \end{array}\right]=\left[\begin{array}{cccc} M_{11} & M_{12} & M_{13} & M_{14}\\ M_{21} & M_{22} & M_{23} & M_{24}\\ M_{31} & M_{32} & M_{33} & M_{34}\\ M_{41} & M_{42} & M_{43} & M_{44} \end{array}\right]\cdot \left[\begin{array}{c} I_{i}\\ Q_{i}\\ U_{i}\\ V_{i} \end{array}\right]$$

where $I_{i},Q_{i},U_{i},V_{i}$ and $I_{o},Q_{o},U_{o},V_{o}$ represent the Stokes vectors of the incident and outgoing light, respectively. $M_{ij}$ is a matrix element.

By systematically modulating the polarization states generated by the PSG (e.g., adjusting the combination angles of polarizers and retarders) and simultaneously measuring the PSA outputs, the complete 16-element Mueller matrix can be acquired. Compared to conventional ellipsometry, which measures only Ψ and Δ, MME enables the capture of complex optical behaviors of the sample, such as anisotropy and depolarization effects.

**Figure 3:**
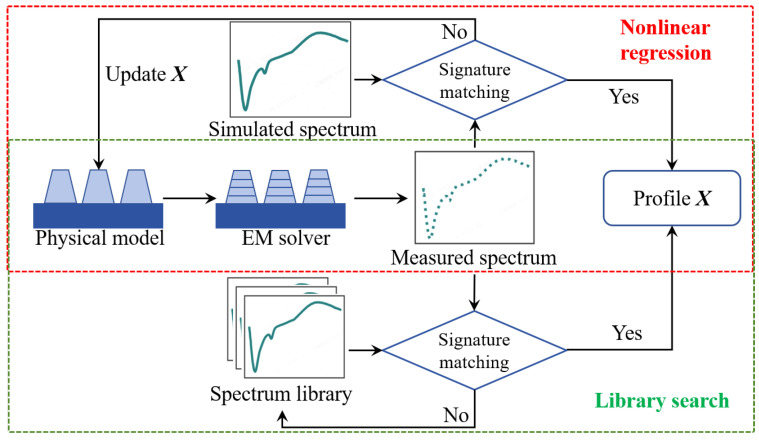
Workflow for solving inverse problems in optical metrology: red box (nonlinear regression using iterative optimization); green box (library search with pre-computed spectra); arrows indicate parameter adjustment and matching steps [4].

As illustrated in Figure 3, the measured Mueller matrix must be inverted to determine the sample’s physical parameters (e.g., linewidth, sidewall angle, film thickness). This inversion process comprises two main steps:

Electromagnetic (EM) solver: A parameterized structural model is established (e.g., describing a trapezoidal grating using parameters like height, top linewidth, and sidewall angle). Rigorous electromagnetic simulation methods, such as Rigorous Coupled-Wave Analysis (RCWA), the Finite Element Method (FEM), or the Finite-Difference Time-Domain (FDTD) method, are employed to solve Maxwell’s equations and compute the simulated Mueller matrix $M_{ij}^{calc}\left(X,\lambda_{n}\right)$, where X is the vector of parameters to be determined.
Inverse Problem Optimization: The parameters are solved by minimizing the residual between the measured matrix $M_{ij}^{meas}\left(\lambda_{n}\right)$ and the simulated matrix $M_{ij}^{calc}\left(X,\lambda_{n}\right)$. The objective function is defined as a weighted sum of squared differences:
  (2) $$\chi^{2}\left(X\right)=\sum_{i,j=1}^{4}\sum_{n=1}^{N}\frac{w_{ij}}{\sigma_{ij,n}}{\left[M_{ij}^{meas}\left(\lambda_{n}\right)-M_{ij}^{calc}\left(X,\lambda_{n}\right)\right]}^{2}$$
  where $w_{ij}$ represents the weights for each matrix element, $\sigma_{ij,n}$ is the measurement variances, and *N* represents number of wavelengths. Classical optimization algorithms for solving this inverse problem include nonlinear regression and library search.

As indicated by the red box in the Figure 3, nonlinear regression employs iterative algorithms like Levenberg–Marquardt to adjust X until convergence is achieved. The advantage of this approach is its flexibility in adjusting parameters. However, it is sensitive to the initial guess and computationally intensive due to the repeated calls to the forward simulation model during iterations. The green box in the Figure 3 represents the library search method. This technique pre-computes a library of simulated spectra across the parameter space. The solution X is determined by finding the library entry whose simulated spectrum best matches the measurement (nearest neighbor). Although building the library requires offline computation, the real-time solution is fast and avoids local minima, making it suitable for online measurement. Interpolation techniques (e.g., polynomial interpolation fitting the error surface) are often applied to the best-matching library points to enhance accuracy.

## 3. AI-Enabled Optical Metrology

### 3.1. Overview of AI Applications in Optical Metrology

Throughout the advancement of science and technology, each breakthrough in AI has generated significant ripples within both academic research and industrial applications. This is particularly evident in the interdisciplinary field of optical spectroscopy, where the trajectory of AI evolution is clearly delineated by trends in research publication outputs.

Following over a decade of skepticism towards Artificial Neural Networks (ANNs) during the 1970s, the 1980s witnessed substantial theoretical developments. A pivotal breakthrough occurred in 1989 when Hornik et al. demonstrated that Multilayer Perceptrons (MLPs) possess universal approximation capabilities; through activation functions like Sigmoid, they can approximate any complex function with arbitrary accuracy, establishing computational equivalence to Turing machines [22]. This finding fundamentally resolved the doubts about neural network expressivity raised earlier by Minsky and others. This breakthrough subsequently triggered a surge in ANN research. In 1993, Richard Krukar pioneered the application of ANNs to optical spectroscopy by demonstrating their effectiveness in classifying semiconductor wafer profiles from diffraction-based measurements [23], followed by growing interest from researchers in this domain.

However, technological limitations soon emerged: constrained by the computational power of the era (primarily single-core CPUs, lacking specialized acceleration hardware), training deep neural networks faced challenges such as vanishing gradients and excessive computation time. Furthermore, data acquisition techniques for realistic scenarios were underdeveloped, leading to a scarcity of large-scale datasets. After 2000, research enthusiasm for ANNs cooled, publications entered a plateau phase, and some scholars shifted focus to shallow learning models like Support Vector Machines (SVMs).

The 2012 ImageNet Large-Scale Visual Recognition Challenge marked a turning point in AI development. The Convolutional Neural Network (CNN) developed by Hinton’s team reduced the classification error rate to 15.3%, significantly outperforming the 26.2% error rate of traditional methods and heralding the advent of the deep learning era [24]. From 2013 onwards, Deep Neural Networks (DNNs), leveraging their powerful feature extraction capabilities, achieved breakthroughs in domains like image recognition and speech processing. Concomitant improvements in computational power (maturation of GPU parallel computing, release of Nvidia’s Kepler architecture) and an explosion in available data (massive data accumulation during the internet era) provided fertile ground for deep learning. Consequently, the volume of AI research publications began to grow exponentially, experiencing an average annual growth rate of approximately 40% between 2013 and 2018.

**Figure 4:**
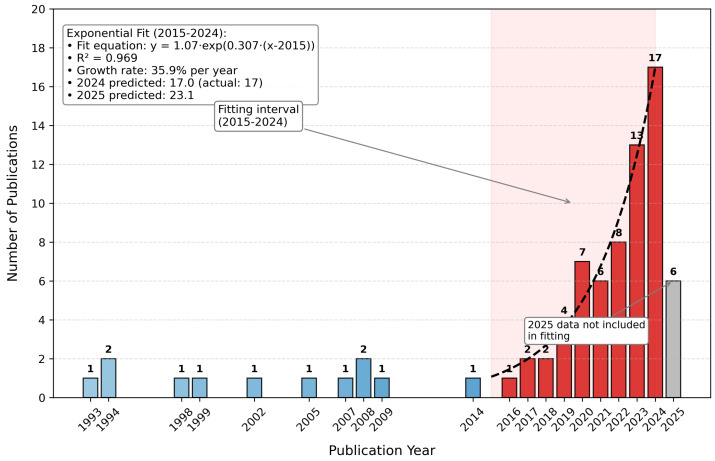
Exponential growth in AI–optical metrology research: X-axis (year from 1993–2025); Y-axis (number of publications); trend line shows rapid increase post-2015, driven by advancements in deep learning and hardware acceleration.

The penetration of AI technology into optical spectroscopy commenced around 2015. In the subsequent years, its influence steadily accumulated. As shown in Figure 4, starting in 2018, publication data reveals a clear exponential growth trajectory (e.g., rising from 2 publications in 2018 to 17 in 2023; while 2025 data is incomplete, the upward trend remains robust).

The proliferation of specialized hardware like GPUs and TPUs, combined with accessible cloud computing resources, has dramatically lowered the barrier to training complex models. Simultaneously, the widespread adoption of high-precision scatterometers has generated massive volumes of high-quality scattering data, providing the indispensable “fuel” for training robust, high-accuracy AI models. Emerging paradigms such as PINNs and transfer learning tightly integrate knowledge from physical models (e.g., Maxwell’s equations) with data-driven approaches [25,26]. This fusion enhances model interpretability and generalization capability, making them particularly suitable for tackling highly nonlinear, high-dimensional scattering problems. Cross-disciplinary research integrating optical spectroscopy and AI is now entering the mainstream.

### 3.2. Forward Surrogate Models

As established earlier, the core task of optical spectroscopy involves solving an inverse problem. Traditional nonlinear regression algorithms suffer from high computational complexity and insufficient real-time performance, making them inadequate for online metrology requirements. While the library search method offers computational speed advantages, its accuracy is inherently limited by grid density. Although interpolation optimization algorithms can improve precision to some extent, they demand massive storage resources due to the requirement of storing vast libraries of simulated spectra.

To overcome these limitations and synthesize the strengths of both nonlinear regression and library search—namely, achieving light computational weight, high speed, and high precision—researchers have turned their attention to neural network technology.

**Figure 5:**
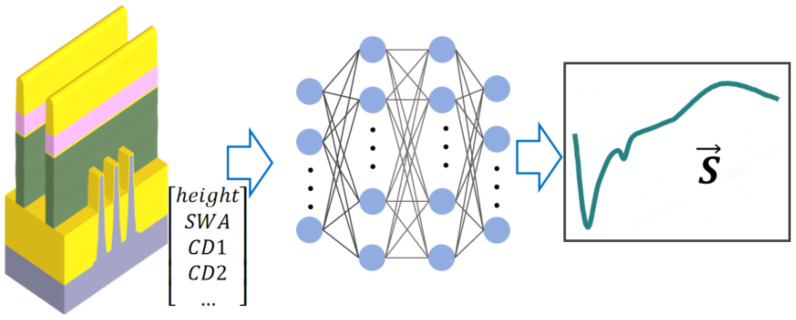
Neural network-based forward surrogate model: input (geometric parameters, e.g., height, top linewidth); processing (neural network trained on EM-simulated data); output (predicted spectra, e.g., Mueller matrix elements). Once trained, this surrogate model directly substitutes the pre-computed library (the green module in Figure 3) within the library search method.

Within advanced semiconductor process technology, precise optical metrology of HAR nanostructures presents significant challenges for traditional electromagnetic solvers, particularly concerning computational efficiency and real-time response capabilities. Researchers are actively developing surrogate models based on neural networks (NNs) that establish mappings between geometric parameters and corresponding spectra, aiming to overcome the challenges associated with inverse problem solving in spectroscopy-based analysis (as depicted in Figure 5). These studies exhibit a significant methodological commonality:

(1) Data-Driven Paradigm: They systematically uncover complex nonlinear mapping relationships between the geometric parameters of nanostructures (including critical dimensions, sidewall tilt angles, and other key parameters) and their optical responses (such as Mueller matrix elements and reflectance spectrum distributions) using a data-driven machine learning approach.

(2) Quality Simulation Data: They rely on generating high-fidelity synthetic datasets via an EM solver to train the models.

(3) Experimental Validation: They incorporate experimental validation to assess the metrological accuracy of the surrogate models.

The core scientific objective of this research is to enhance the efficiency, robustness, and real-time feedback capability of multi-parameter cooperative metrology. This advancement aims to provide data-driven decision support for the precise control of semiconductor manufacturing processes.

Mudide et al. [27] integrated a neural network with MME to construct a forward mapping model from the geometric parameters of HAR structures to their spectra. Their technical approach consisted of: (1) first generating synthetic data via RCWA to train a ANN predicting Mueller matrix spectra; (2) subsequently freezing the network weights; and (3) finally performing gradient descent-based inversion on measured spectra to achieve rapid extraction of key parameters like grating tilt angle. Mudide et al. [27] demonstrated that this method agreed with Small-Angle X-ray Scattering (SAXS) results for Critical-Area-Target (CAT) grating measurements. Crucially, the time required for full-wafer tilt angle mapping was reduced from hours required by traditional methods to mere seconds.

The key technological advantages lie in non-destructiveness and real-time capability—enabling direct measurement after Deep Reactive-Ion Etching (DRIE) without sample thinning, making it highly suitable for in-line production monitoring. Furthermore, replacing RCWA with the ANN surrogate model achieves a three-orders-of-magnitude improvement in gradient calculation efficiency, facilitating rapid scanning of large parameter spaces. However, a significant limitation stems from its reliance on RCWA-simulated training data [27]: if unmodeled factors (e.g., sidewall roughness) exist in actual samples, errors in spectral prediction propagate directly into the inversion results. Additionally, the high information content of Mueller spectra necessitates models with high-dimensional outputs, requiring substantial data and extensive training time for the surrogate model to converge.

Liu et al. [28] proposed the “PCA Dimensionality Reduction–Neural Network Surrogate–Iterative Optimization” framework. This approach employs Principal Component Analysis (PCA) to compress the dimensionality of Mueller matrix spectra from 1455 down to 204 dimensions. A fully connected NN then maps parameters to these lower-dimensional feature spectra. Finally, the LM algorithm iteratively optimizes the parameters by matching the measured and predicted spectra. Experiments showed that this method achieved a 30-fold speed-up in grating parameter extraction compared to library search. Moreover, its robustness in noisy instrument environments significantly outperformed End-to-End Deep Neural Networks (DEDNN)—reducing inversion error by an order of magnitude relative to DEDNN.

The technical breakthrough lies in the synergistic combination of dimensionality reduction and iterative optimization: PCA filters redundant spectral information, concurrently reducing NN training data requirements and mitigating sensitivity to noise; and iterative optimization compensates for model mismatch between simulation and reality through dynamic adjustment of initial parameter values. Nonetheless, limitations remain: dimensionality reduction carries inherent feature loss risks—the number of principal components (e.g., 204) must be determined empirically, potentially obscuring critical spectral features; and the iterative optimization’s convergence relies heavily on the initial guess, risking convergence to local minima for highly asymmetric structures.

Beyond direct involvement in the metrology process, surrogate models also find applications in sensitivity analysis frameworks.

Despite the limitations discussed earlier regarding dimensionality reduction and iterative optimization (e.g., the risk of feature loss and initial value dependency), ANN surrogate models demonstrate significant advantages in overall performance. As shown in Table 1, compared to traditional nonlinear regression and library search methods, ANN surrogate models achieve a superior balance across three key dimensions: computational speed, measurement accuracy, and storage efficiency. They maintain sub-nanometer accuracy (MAE < 0.1 nm) while reducing single-parameter extraction time from hours to seconds and entirely eliminating the need for massive spectral libraries.

Meng et al. [29] utilized an NN surrogate within a Density-Based Sensitivity Analysis (DBSA) system to quantify the statistical impact of structural parameters on spectral responses. The innovation lies in introducing the PAWN (derived from the authors names) index to measure spectral distribution changes induced by parameter variations [30]. Combined with iterative Latin Hypercube Design (LHD) to optimize measurement configurations (e.g., incident wavelength, azimuthal angle), this framework enables the collaborative sensitivity assessment of dual-pattern gratings involving over eight parameters. For instance, analyzing the spectral sensitivity of Top Critical Dimension (TCD) and Sidewall Angle (SWA) at different azimuthal angles guides optimal measurement angle selection.

The core value of Meng et al.’s framework [29] is its capability to capture multi-parameter interaction effects—nonlinear coupling phenomena that conventional Local Sensitivity Analysis (LSA) fails to characterize can be precisely quantified via the NN surrogate model combined with variance decomposition. However, computational complexity impedes real-time application: iterative sampling necessitates generating $∼\;10^{4}$ samples, leading to pre-computation times reaching 34 h. Furthermore, noise in experimental spectra can significantly amplify errors in computed distribution distances (e.g., Kolmogorov–Smirnov distance), demanding additional data cleaning and preprocessing.

Current technological bottlenecks primarily center on three interconnected challenges. First, significant spectral oscillations occur in structures with high aspect ratios, while large pitch structures exhibit sharp resonant peaks in their spectra; these complex physical phenomena create highly nonlinear relationships that make it difficult for surrogate models to converge during training. This difficulty is compounded by, secondly, the inherent distributional discrepancies between simulated training data and real-world measurement conditions, meaning models optimized on idealized simulations struggle to generalize to noisy, complex experimental data. Finally, achieving robust generalization across high-dimensional parameter spaces remains a major hurdle, as models often fail to accurately predict performance or behavior for design configurations significantly different from those seen during training.

### 3.3. Inverse Prediction Models

While forward surrogate models leverage the strengths of both nonlinear regression and library search algorithms, they necessitate a priori knowledge of a parametric physical model for the target structure. Training data is subsequently generated via simulation, with an inverse problem mapping then implemented using neural network algorithms. This entire process remains dependent on both the underlying physical model and iterative optimization.

**Figure 6:**
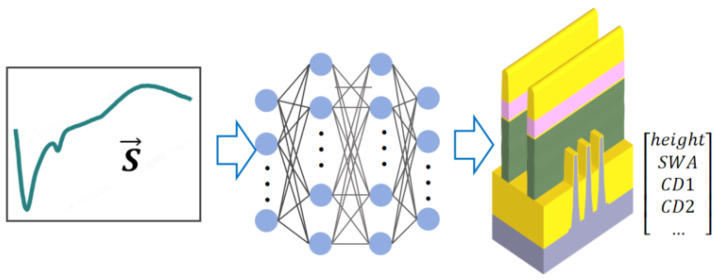
Inverse prediction model framework. (**Left**): Input vector $\vec{S}$ (measured spectral signal). (**Center**): Neural network mapping spectra to parameters. (**Right**): Output parameters.

Constructing a inverse predict model that maps spectra directly to CDs offers an intuitive approach for parameter retrieval(shown in Figure 6) [31,32,33,34,35,36,37,38,39,40,41,42,43]. Pioneering research in 1998 demonstrated the use of diffraction order intensities as neural network inputs to predict grating CDs [31]. Limited by early computational power, initial models employed constrained input feature dimensions. For instance, Wei et al. utilized only six sets of multi-wavelength scattering efficiencies as input [35]. By 2023, however, significant advancements were evident, with Fu et al. successfully incorporating the full-spectrum data of all 16 Mueller matrix elements into the input dimensionality, enabling more comprehensive feature extraction [36].

Significant progress has been reported regarding the speed and accuracy achieved through direct inverse predict models parameter mapping. For example, Sabbagh et al. employed an XGBoost model capable of completing CD metrology for a 300 mm wafer within minutes—a reduction in computational time exceeding 10-fold compared to traditional optimization algorithms, dramatically enhancing efficiency [37]. Similarly, Fu et al. utilized a ResNet neural network for grating parameter prediction, slashing computation time from 541 s using conventional library search to merely 4 s, representing an acceleration of approximately 135 times [36]. This breakthrough provides robust support for the realization of real-time process monitoring. In terms of precision, the XGBoost model employed in Sabbagh’s work for nanostructure CD prediction achieved errors below 5 nm, with deviations from SEM measurements achieving less than 3%, demonstrating exceptional accuracy [37]. These findings are corroborated by other studies [35,40]. Furthermore, Bahrenberg et al. demonstrated results obtained using Gage R&R, surpassing those from SEM [38]. Kfoury et al. trained an ANN model on spectroscopic ellipsometry data, achieving remarkably low prediction errors (0.3% for silver nanoparticle volume fraction and 4.8 nm for thickness) [42]. Collectively, these studies demonstrate that neural networks, via data-driven direct mapping mechanisms, circumvent the time-consuming iterative optimization inherent to traditional physics-based models. By leveraging large-scale data training and deep feature extraction capabilities, they overcome the limitations of conventional methods in both speed and accuracy, offering an efficient and precise technical pathway for fields like nanometrology and semiconductor process control.

The core advantage of the inverse prediction model lies in its independence from parametric physical models, enabling the direct inference of critical dimensions solely from scattering information. For instance, studies [39,42] directly mapped spectral data to CDs, eliminating the need to construct RCWA, FDTD, or FEM physics-based models, thereby reducing manual modeling costs. Similarly, Fu et al. utilized Mueller matrix images to directly infer grating SWA, avoiding assumptions about the grating shape required in traditional methods [36]. This liberation from dependence on parametric physical models confers substantial model flexibility and enhances the potential for application expansion. Table 2 lists representative studies utilizing inverse prediction models to map general information from scattering signals.

Liu et al. use the sketch-guided neural network (SGNN), embedding sketch guidance into deep learning architectures. Centered on generic contour models, SGNN eliminated dependency on predefined geometric templates for nanostructure contour reconstruction. Trained on 60,000 simulated and experimental datasets, SGNN directly maps optical features (e.g., Mueller matrix spectra) to structural contours without complex parametric physical models, significantly enhancing generalization for unknown structures [44].

Some studies focused on parameter measurements in industrial contexts [45,46,47,48,49,50]. Leveraging AI, they achieved precise predictions of key metrics including local CD uniformity, electrical properties of metal lines, via tilt angles, defect densities, and alloy stoichiometry. Similar work has utilized not only scattering spectra as input but also incorporated optical emission spectroscopy (OES) for predicting metrics such as etch rate [54,55]. The studies have also replaced time-consuming multi-step traditional workflows with data-driven nonlinear mappings between optical signals and target parameters. Collectively, these studies reveal the core advantages of inverse predict models in optical spectroscopy: by directly mining latent patterns from real measurement data, they circumvent the complex physical model assumptions and extensive simulated data preparation required by traditional approaches. Conventional methods rely on precisely parameterized physical models to describe optical scattering processes, demanding significant time for model construction and simulation validation. In contrast, AI models leverage powerful feature extraction and pattern recognition capabilities to adaptively learn complex relationships between optical signals and target parameters, substantially simplifying the measurement workflow while enhancing efficiency and flexibility. Particularly in scenarios challenging for conventional methods—such as analyzing complex structures or unknown defect types—inverse prediction models demonstrate superior adaptability and generalization, paving new pathways for optical spectroscopy and propelling it toward intelligent and efficient advancement.

### 3.4. Overcoming Real-Spectral-Data Challenges: PINN Paradigms

To address the challenges of high cost and limited availability of real spectral data, recent research has focused on embedding physical prior information into AI models [56]. Three typical paradigms have been developed, which significantly reduce the models’ dependence on real data and enhance their physical consistency.

The first paradigm is physical constraint regularization, which involves introducing physical constraint terms into the loss function to ensure that the output of the AI model adheres to fundamental physical laws. For example, in spectroscopic ellipsometry, the energy conservation constraint $N^{2}+C^{2}+S^{2}=1$ for ellipsometric parameters is transformed into a regularization term that is optimized jointly with the data fitting term [25]. The loss function is shown as follows:

(3) $$L=\sum {\left(I^{meas}-I^{calc}\right)}^{2}+Spa\left(\hat{n},\hat{c},\hat{s}\right)+Phy\left(\hat{n},\hat{c},\hat{s}\right)$$

where $I^{meas}$ represents the measured spectrum and $I^{calc}$ represents the simulated spectrum. $Spa(\hat{n},\hat{c},\hat{s})$ represent sparsity prior, and the physics-informed regularization as follows:

(4) $$Phy\left(\hat{n},\hat{c},\hat{s}\right)={∥N^{2}+C^{2}+S^{2}-1∥}_{2}^{2}$$

This approach has been validated in channel spectroscopic ellipsometry (CSE). Even in the presence of systematic errors, it can maintain the root-mean-square error (RMSE) of ellipsometric parameter measurements within the range of 0.0051–0.0247, significantly outperforming traditional Fourier reconstruction methods.

The second paradigm is the hybrid training with physical models [57]. It utilizes physical models to generate a large-scale synthetic spectral dataset, which is then combined with a small amount of real data to form the training set. The contribution of physical information can be adjusted by the weight in the loss function, as shown in the following:

(5) $$L=w_{1}I^{meas}+w_{2}I^{calc}$$

where $I^{meas}$ represents loss caused by measured spectrum, $I^{calc}$ represents loss caused by simulated spectrum; and $w_{1}$ and $w_{2}$ are weights.

In the prediction of the thickness of transparent conductive oxides (TCOs), for instance, CNNs trained with synthetic data generated by physical models can achieve sub-second reconstruction of full-wafer thickness maps using only 5–10 real reference points, with a thickness deviation of less than 8 nm compared to SEM measurements. In semiconductor etching process monitoring, after mixing a large number of simulated data with a small amount of real spectra, the model can achieve a measurement accuracy of 0.95 Å (3σ) for buried structures, approaching the accuracy level of traditional physical modeling [58].

The third paradigm is pre-training with transfer learning. It first employs physical models to generate millions of synthetic data for pre-training neural networks, followed by fine-tuning with a small amount of real data (usually ≤20 groups) [26]. In the quality control of diffraction gratings, for example, a MLP pre-trained with diffraction spectra simulated by physical models under various parameters can be fine-tuned using real grating data. After fine-tuning, the measurement deviations for period, linewidth, and height are reduced to 1.3 nm, 4.3 nm, and 2.6 nm, respectively, consistent with SEM measurements within the uncertainty range. A similar idea can be found in Kim’s study [59].

Embedding physical information brings several significant advantages. Firstly, it greatly improves data efficiency, reducing the requirement for real data from hundreds of groups in traditional methods to dozens or even eliminating the need for real data entirely. Secondly, it ensures physical consistency, preventing model outputs from violating physical laws. Thirdly, it enhances generalization ability. In cross-process scenarios, the error fluctuation of AI models guided by physical information is less than 15%, far lower than that of pure data-driven models. These paradigms provide efficient and accurate solutions for fields such as optical metrology and semiconductor manufacturing, driving the evolution of AI models from “data fitting” to “physical understanding”.

### 3.5. Multi-Stage Network Architecture

A single AI model often struggles with increasingly complex optical spectroscopy measurements of semiconductor structures. Consequently, a growing body of research proposes solutions employing multiple AI models or hybrid approaches combining AI with traditional algorithms.

The dual-stage neural network division-of-labor mechanism has emerged as the dominant paradigm for solving complex inverse problems (Jung et al. even employed a three-stage network [60]). This strategy follows a “contour classification–parameter regression” two-step process. First, a classification network identifies the geometric profile of the nanostructure. Then, a regression network performs high-precision parameter extraction. For instance, the architecture achieved a grating profile recognition accuracy of 96.9% and kept the MAE for structural parameters below 0.1 nm [61]. Multi-branch architectures further optimize parameter decoupling. Digraci et al. utilized independent branches for CDs prediction, reducing the CDs prediction error from 3.5% to 2.9% and effectively mitigating error amplification caused by parameter coupling in a single AI model [62].

The core innovation of such architectures lies in decomposing a global optimization problem into localized feature extraction tasks, significantly constraining the solution space through hierarchical processing. As an example, a two-step method first matches Mueller matrix library data using the LM algorithm and then employs a neural network for parameter prediction within a localized region [63]. This achieved sub-nanometer level accuracy (MAE < 0.1 nm) for the width and height of 1D nano-gratings. However, this method exhibits strong dependency on the training data distribution. When structural parameters exceed the preset range, systematic bias may arise; experiments showed recognition errors for non-standard contours as high as 15% [64].

**Figure 7:**
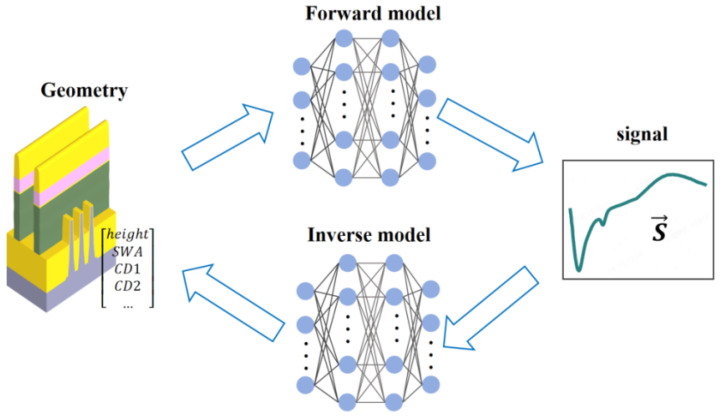
Integrated framework combining inverse and forward models: top loop (inverse network mapping spectra to parameters); bottom loop (forward physical verification using EM solvers); weighted loss function (Equation (6)) balances errors.

Some studies combine the forward and inverse AI models introduced in Section 3.2 and Section 3.3, as shown in Figure 7. They commonly adopt a closed-loop architecture of “inverse network generation + forward physical verification” to tackle optical spectroscopy inverse problems [65,66,67]. The inverse networks map measured data (e.g., ellipsometric parameters, reflection spectra) to parameter solutions using neural architectures like SRUM or quantized CNNs, integrating physical constraints (e.g., energy conservation) to avoid ambiguity. Forward networks then validate these solutions via physical models (e.g., Fresnel equations, FDTD simulations), calculating discrepancies between predicted and measured data. Weighted loss functions can be commonly expressed as follows:

(6) $$L=w_{fwd}L_{fwd}+w_{inv}L_{inv}$$

where $L_{fwd}$ and $L_{inv}$ are the loss terms for the forward and inverse models, respectively, and $w_{fwd}$ and $w_{inv}$ are their corresponding weights. They balance errors in both directions, and iterative optimization ensures physically reasonable and accurate solutions. This unified framework enables efficient, robust parameter retrieval across diverse applications.

Hybrid strategies combining neural network initialization with traditional algorithm iteration demonstrate unique advantages in industrial settings [39,68]. First, a mapping of the ANN from the spectrum to the key parameters is constructed using simulation data. Then, the trained ANN processes the measured reflectance spectrum to provide an initial estimate of the geometric parameter vector. After that, the LM algorithm is used for iterative optimization until the simulated reflectance spectrum matches the measured data [68]. The improved metrology achieves highly accurate measurement results and fast computation speed through the ANN/LM combined parameter extraction method. The core advantage of this framework is its balance between computational efficiency and global optimization capability: the neural network handles complex nonlinear mappings, while the traditional algorithm corrects local deviations.

Robert et al. employed a dual neural network architecture (ANN1 for parameter regression + ANN2 for variance estimation) to construct error confidence intervals. This narrowed the local error range for silicon grating profile reconstruction by 50%, providing probabilistic assessment for process quality control [33]. Current research has achieved breakthroughs in measuring nano-gratings, deep trenches, and TSVs. In semiconductor manufacturing, the methods described in [39,63] enabled CDs monitoring for nano-grating etching and TSV filling processes, respectively, with measurement errors controlled at the 0.1–1 nm level. In optical thin films, the Terahertz Ellipsometry method enabled rapid parameter extraction for organic films using single-frequency measurements [65].

### 3.6. Challenges

Most existing studies focus on improving the accuracy of memory and logic chips, while few address the simulation slowdown caused by high-aspect-ratio memory chips and high-power periodic chips in surrogate modeling scenarios. Only Ahn et al. proposed using CNNs to replace eigenvector solvers for accelerating RCWA simulations [69]. For power and memory chips exhibiting sharp spectral peaks and intensive oscillations, constructing surrogate models requires significantly more spectral data. The substantial time cost of simulating individual spectra for such structures can demand weeks, presenting a critical challenge for data generation. Furthermore, research on few-shot learning remains insufficient beyond physics-informed approaches [70,71]. Academic studies typically offer theoretical depth but lack practical data and application scenarios, whereas industrial research covers broad topics with rich scenarios but limited depth. Intense competition in the semiconductor industry leads to strict confidentiality of measurement data, making public dataset construction nearly impossible. Bridging the data gap between industry and academia warrants significant effort and innovative approaches.

## 4. PMISH’s AI Architecture and Examples

**Figure 8:**
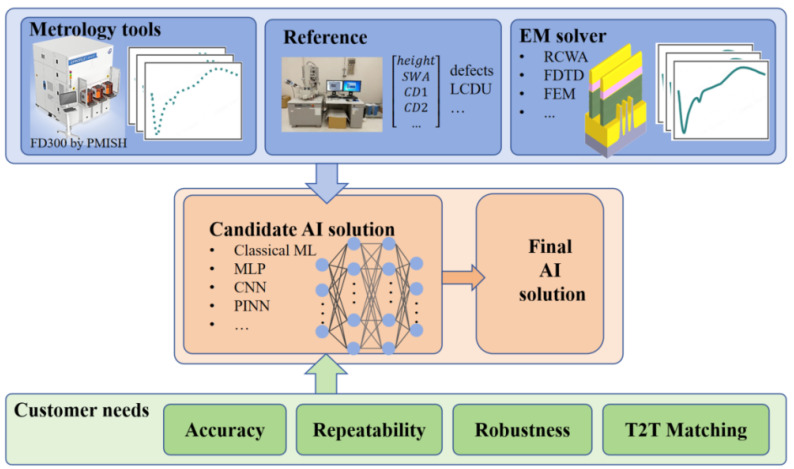
PMISH’s J-profiler AI architecture: top layer (data input—simulation data, measurement data, and reference data, in flexible combinations); bottom layer (requirement input—customer specifications, e.g., accuracy or T2T matching); middle layer (AI engine—automatically selects algorithms based on inputs to generate final AI solutions).

PMISH has developed a software named J-profiler 5.0 that integrates physics-based RCWA with AI algorithms. This integration provides high-precision modeling capabilities for optical spectroscopy. J-profiler’s advanced 3D modeling engine supports RCWA simulations of arbitrary 3D periodic structures. Furthermore, the AI capabilities reviewed earlier have also been incorporated into J-profiler; the architecture is shown in Figure 8. Its core algorithm library encompasses numerous AI algorithms. Depending on the availability of customer data and specific requirements, the software enables manual or automatic selection of appropriate AI algorithms to generate tailored AI solutions.

**Figure 9:**
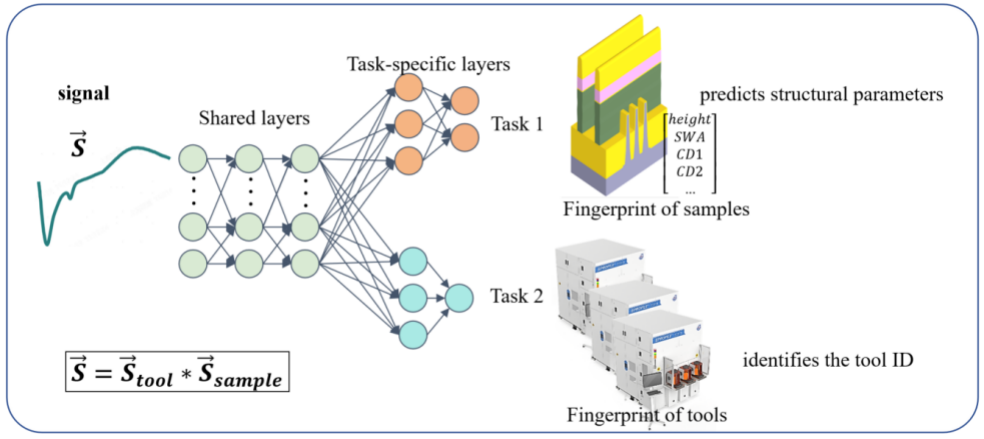
Our Multi-Task Learning (MTL) framework for T2T matching: input (spectral response); Branch 1 (regression network for parameter prediction); Branch 2 (classification network for tool identification); output (decoupled parameters and tool signatures).

Key customer requirements addressed include metrology accuracy, precision, robustness, and T2T matching. In this context, accuracy denotes the deviation between measurements obtained by PMISH’s metrology tools and customer-provided reference (“golden”) values; precision refers to dynamic and static repeatability; and T2T matching signifies measurement consistency across different metrology tools. While T2T matching is a critical industry concern, it receives scant attention in academic studies. The few papers mentioning T2T matching are primarily promotional publications by relevant companies and typically lack substantive technical detail [72]. A method based on stochastic polynomial wavelength calibration (s-PWC) has been developed to address the T2T matching issue, but it appears to have overly one-sided considerations [73]. We therefore selected T2T matching as an exemplar to demonstrate the capabilities of PMISH’s next-generation J-profiler metrology technology. At a specific semiconductor manufacturing customer site utilizing multiple PMISH OCD metrology tools, most CDs exhibited strong measurement consistency across tools. However, for CDs exhibiting low sensitivity to optical signals within certain complex nanostructures, T2T consistency degraded. This inconsistency necessitated AI intervention. A measured optical signal $\vec{S}$ inherently contains two coupled components, the sample’s optical response ${\vec{S}}_{sample}$ (sample signature) and the tool’s intrinsic response ${\vec{S}}_{tool}$ (tool intrinsic signature). Theoretically, decoupling the tool’s intrinsic signature from the measurement signal could achieve consistent results across all tools. We designed an algorithm framework based on Multi-Task Learning (MTL), as shown in Figure 9. The neural network input is the spectral response signal. The output has two branches: a regression network predicting the problematic, low-sensitivity parameters; and a classification network identifying the specific metrology tool that captured the input signal.

**Figure 10:**
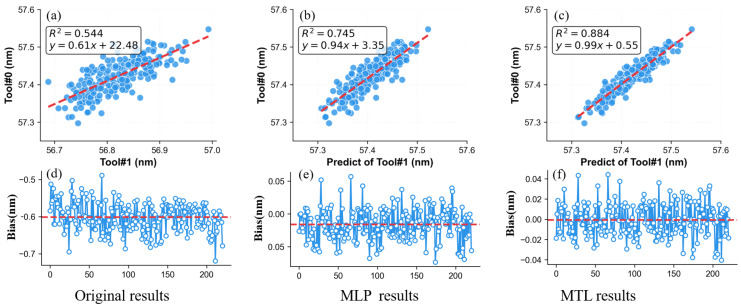
Performance of our MTL framework comparison for T2T matching. (**a**–**c**) represent the correlations of the original measurements, standard MLP regression network, and MTL network for Tool#0 and Tool#1, respectively; (**d**–**f**) show their respective biases. Our MTL framework reduces mean bias to near-zero and improves consistency.

Our MTL framework inherently forces feature separation. The classification branch learns to identify the tool’s intrinsic signature. In our industrial case study, the regression branch becomes primarily influenced by the sample signature, and our PMISH’s J-profiler 5.0 software mitigated tool-induced variation and solved the T2T matching problem. As shown in Figure 10, the results indicate that raw measurements between Tool #0 and Tool #1 showed poor consistency (coefficient of determination, R^2^ = 0.54) and a large systematic bias. Employing a standard MLP regression model without signature decoupling significantly improved T2T matching (R^2^ increased to 0.75) and reduced bias, bringing the average close to zero. Applying the MTL framework to decouple the signatures yielded further substantial improvement: R^2^ reached 0.88, the bias range was reduced to 32% of its initial magnitude, and the mean bias was virtually zero.

The MTL framework effectively decouples the sample signature from the tool’s intrinsic signature within optical metrology signals. This capability directly addresses and significantly enhances T2T matching performance.

## 5. Summary and Outlook

This study comprehensively examines the current state and future trajectory of AI-powered optical spectroscopy within semiconductor advanced manufacturing metrology. The integration of AI with spectroscopy has achieved significant milestones: forward surrogate models enhance multi-parameter co-measurement efficiency by establishing accurate parameter-spectrum mappings; inverse prediction models overcome limitations of traditional physics-based parameterization, enabling direct reconstruction of CDs from spectroscopy spectra; PINNs reduce reliance on costly measurement data while ensuring physical consistency; and multi-level network architectures demonstrate high precision in solving inverse problems for complex structures and are successfully used in process monitoring for various typical devices. Practices at PMISH confirm AI’s effectiveness in resolving real-world industrial challenges like T2T matching deviations.

However, significant challenges remain. Data-wise, distributional discrepancies between simulated and measured data hinder model transferability, while data scarcity limits generalization for tasks like rare defect detection. Algorithm-wise, current methods struggle with the diverse measurement needs of heterogeneous integrated devices spanning sub-micron to millimeter scales; deploying lightweight models on edge-devices risks accuracy loss, and balancing real-time performance with measurement accuracy is unresolved.

Future research should prioritize: (1) Deepening the fusion of data-driven and physics-based models, exploring efficient cross-structure knowledge transfer via reinforcement learning and transfer learning. (2) Developing unified multi-scale characterization algorithms for cross-scale semiconductor device measurements. (3) Designing low-complexity, high-accuracy neural architectures, leveraging GPU acceleration to enhance real-time performance without compromising accuracy. Furthermore, strengthening industry-academia collaboration is crucial to accelerate the translation of lab innovations into industrial applications, establishing an intelligent, high-precision metrology framework for advanced semiconductor manufacturing.

## Figures and Tables

**Table 1:** Comparison of computational performance for different inverse problem-solving algorithms.

Method	Speed	Accuracy	Storage
Nonlinear Regression	Slow	High	Small
Library Search	Fast	Low	Large
ANN Surrogate	Fast	High	Small

**Table 2:** Examples of work utilizing inverse predict models for scattering signals mapping.

Target Parameters	Core Algorithm and Models	Results and Discussion	Limitations
Nanostructure profiles [44]	Sketch-guided neural network (SGNN) integrated with generic profile model, CNN architecture with profile smoothing	SGNN achieves MAE < 1.5 nm for rectangular/trapezoidal gratings. Experimental comparison shows MSE as low as $1.32\times 10^{-3}$ compared to nonlinear regression, outperforming traditional deep learning in generalizability	The universal profile model remains a parametric straitjacket. No fixed geometric abstraction can encapsulate the infinite variability of nanofabrication physics
Local critical dimension uniformity (LCDU), critical dimension (CD) [45]	Supervised learning with PCA, trained using CD-SEM data as reference	For 44 nm pitch EUV vias, LCDU measurement shows R^2^ > 0.92 with CD-SEM, achieving 40% throughput improvement; supports in situ comparison before and after etching	Sensitive to defect noise under extreme dose/focus conditions; small CD-SEM sample size may introduce statistical bias.
Metal line resistance, capacitance [46]	MLP combined with traditional OCD model, trained on IMEC N14 process data for spectral–electrical property mapping	Resistance prediction shows R^2^ = 0.93, capacitance prediction R^2^ = 0.97, improving 20% accuracy over traditional RCWA models; correlates spacer thickness with defect density	Relies on backend electrical test data labeling; limited transferability to new material systems
Channel hole tilt angles (Tilt-X, Tilt-Y) [47]	Multilayer perceptron (MLP) combined with Mueller matrix analysis, using PCA for dimensionality reduction on 45 wafers’ spectral data	Tilt-X/Y measurements show R^2^ > 0.92 with HV-SEM, 3σ precision < 1.2 nm, enabling 40% throughput improvement for in-line 3D NAND etching monitoring	In HAR structures, optical signals are affected by sidewall shadowing, increasing measurement bias at extreme tilt angles
SiGe nodule defect density, vertical location [48]	Supervised learning (random forest/neural network) fusing scatterometry spectra with CDSEM/TEM image features	For GAA nanosheet structures, defect density prediction accuracy > 95%; correlates spacer thickness with vertical defect distribution (e.g., 40% defects below hardmask)	Limited spectral feature extraction for sub-10 nm nodules; requires high-resolution reference data
CDs, LER [49]	Machine learning regression model (based on Nova SpectraProbe) with Mueller matrix asymmetry analysis	For 32–40 nm pitch EUV resists, CD/LER show R^2^ = 0.995/0.87 with AFM/CDSEM; single measurement replaces four traditional steps	LER/LTR measurements limited by spectral signal-to-noise ratio, with larger errors for small-scale roughness (<2 nm)
Alloy stoichiometry (e.g., x in $Au_{x}Ag_{1-x}$) [50]	MLP (108-dimensional ellipsometric angles Ψ/Δ input, 10-node hidden layer), trained on 30 EDX-spectral pairs	For Au-Ag alloys, x measurement shows $R^{2}$ = 0.92 with EDX; gradient mapping resolution reaches 0.6 mm, identifying compositional gradient trends	Small training sample size (30 pairs); requires re-modeling for ternary alloys (e.g., Au-Ag-Cu)
Defect types [51]	Semi-analytical model (based on TIS theory) +MLP with 121-dimensional spectral input and 10-node hidden layer	For Si grating defects, semi-analytical model achieves MAE < 0.5 nm for interface defects; MLP shows R^2^ = 0.993 for substrate defects, processing at 0.46 ms/run (library search requires tens of thousands of RCWA calculations)	Semi-analytical model fails for complex embedded substrate defects; MLP generalizes poorly to non-sinusoidal substrate defects
Grating profile type [52]	MLP with 36-dimensional spectral intensity ($I_{s}/I_{c}$) input and binary-coded class output	Achieves > 96% classification accuracy for 2 μm periodic gratings, assisting spectroscopy model selection (e.g., trapezoidal model reduces MSE by 40%)	Supports binary classification only, unable to handle mixed profiles (e.g., trapezoidal with rounded corners)
Complex refractive indices (n and k) [53]	Encoder–decoder convolutional neural network (EllipsoNet and C-EllipsoNet), with a loss function combining MSE and 1-PCC	Trained on 450,000 simulated multilayer stacks, achieves median PCC of 0.88 on unseen test data. Predicts n and k for experimental 2D materials ($MoS_{2},MoSe_{2}$, etc.) with reasonable accuracy	Spontaneously learns Kramers–Kronig relations; prediction accuracy for experimental data is slightly lower than for simulated data; performance degrades for structures with fewer material variations in substrates

## Data Availability

The raw data supporting the conclusions of this article will be made available by the authors on request.
